# Changing Strategies of the Retrograde Approach for Chronic Total Occlusion During the Past 7 Years

**DOI:** 10.1002/ccd.24447

**Published:** 2012-04-19

**Authors:** Toshiya Muramatsu, Reiko Tsukahara, Yoshiaki Ito, Hiroshi Ishimori, Seung-Jung Park, Robert Winter, Khaled Shokry, Lefeng Wang, Jiyan Chen, Haichang Wang

**Affiliations:** 1 Division of Cardiology, Saiseikai Yokohama-City Eastern Hospital3-6-1 Shimosueyoshi, Tsurumu-ku, Yokohama-Shi, Kanagawa-Pref., Japan; 2 Asan Medical CenterSeoul, South Korea; 3 Academic Medical CenterAmsterdam, Netherlands; 4 Kobry El Kobba Mulitary HPCairo, Egypt; 5 Beijing Chaoyang HPBeijing, P.R. China; 6 Guangdong Provinnce People's HPGuangzhou City, Guangdong, P.R. China; 7 Xi'jing HPShaanxi, P.R. China

**Keywords:** PCI, CTO, Retrograde approach

## Abstract

**Objective:**

We reviewed the technical changes and results achieved with the retrograde approach since we introduced it 7 years ago.

**Subjects and Methods:**

The subjects were 1,268 patients who were treated for CTO between January 2004 and December 2010. They were investigated with respect to the success rate, the frequency of employing the retrograde approach and its outcome, and other factors.

**Results:**

The retrograde approach was employed in ∼30% of chronic total occlusion (CTO) patients (*n* = 281) and the retrograde guidewire success rate was 81.1%. The kissing wire technique was substituted for the retrograde approach in 126 of the 281 patients, with antegrade crossing of a guidewire being successful in 88 of them (70%). The retrograde approach was combined with the CART and reverse controlled antegrade retrograde tracking (CART) techniques in 22 and 21 patients, respectively. Among 83 patients treated with Corsair catheters, crossing of the CTO was achieved in 63. The overall procedural success rate was 79.7% (224 patients). Complications of the retrograde approach included collateral channel dissection (2.1%), channel perforation (1.7%), CTO perforation (1.7%), and donor artery occlusion (1.1%).

**Conclusion:**

The success rate and safety of the retrograde approach are both satisfactory if the appropriate devices and techniques are selected. © 2012 Wiley Periodicals, Inc.

## INTRODUCTION

Treatment of chronic total occlusion (CTO) by percutaneous transluminal intervention (PCI) has progressed markedly over the past few years. Early and long-term results have improved dramatically because of various modifications of the techniques employed and the advent of new devices [1,2]. Several years ago, the retrograde approach was introduced, in which a guidewire is inserted into the distal end of the CTO from the contralateral side via a collateral channel [3]. In the first report on the retrograde approach, the guidewire was inserted via a bypass graft [4], but a native channel has been employed in most patients more recently. Since we introduced the retrograde approach in 2004, we have made various attempts to improve the devices and techniques used. Here, we review the technical changes and the outcome of the retrograde approach to CTO over the past 7 years.

## SUBJECTS AND METHODS

We treated 1,268 patients with CTO between January 2004 and December 2010. This was a multicenter single-operator study, in which the subjects were patients of our institution or other institutions (including overseas ones) and the principal interventionist was the first author of the present report. We investigated the early success rate, the frequency of using the retrograde approach and its outcome, and various other factors. CTO was defined as occlusion with TIMI grade 0 or 1 blood flow that persisted for at least 6 months. The indications, for PCI of CTO were (1) no anuerysmal changes the left ventricle on echocardiography and (2) successful intervention was considered to be possible by at least two interventional cardiologists. PCI was defined as successful if the residual visual stenosis was less than 20% and antegrade blood flow was TIMI grade 3 or better. Patients with acute myocardial infarction (defined on the basis of a three-fold or greater increase of plasma CK-MB) or occlusion of venous bypass grafts were excluded.

**Fig. 1:**
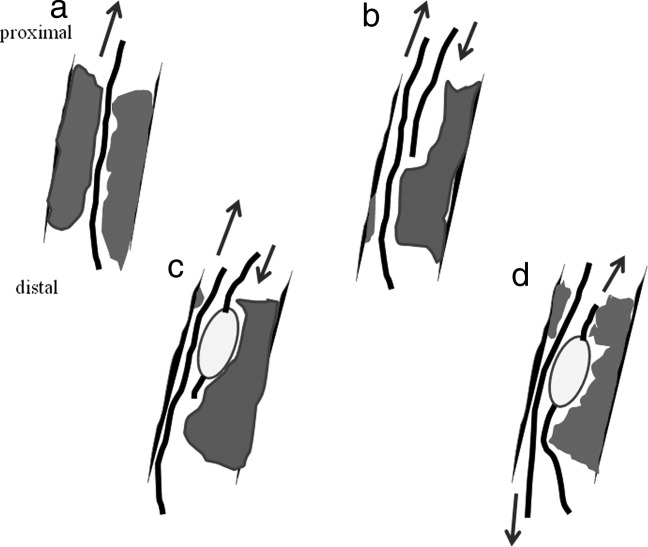
Retrograde CTO crossing techniques. (a) Retrograde wiring, (b) kissing wire technique, (c) Reverse CART technique, (d) CART technique.

PCI was usually performed via the transfemoral approach with 7F sheath and guide catheters. For the antegrade approach, a soft guidewire was tried first and this was switched to a guidewire with a harder tip like a conquest guidewire (Asahi Intecc Co., Japan) if the first attempt failed. For the retrograde approach, Fielder FC guidewires (Asahi Intecc Co., Japan) or the Sion blue guidewire (SJM Co., Japan) were generally used to traverse the collateral channel. If the guidewire passed through the collateral channel successfully, a microcatheter or an over-the-wire balloon system was subsequently inserted. If wiring was unsuccessful, the collateral channel was dilated by the septal channel dilation technique with a 1.25-mm balloon at a pressure of 3 atm. The retrograde guidewire was inserted from the distal end of the CTO to achieve retrograde crossing of the lesion. When a Fielder guidewire could not be passed through the vessel, recanalization of the CTO was attempted with stiffer guidewires in a stepwise fashion, as well as by appropriate use of the kissing wire technique (KWT), CART technique, reverse CART technique, or other methods (Fig. 1). If a channel dilator (Corsair, Asahi Intecc Co., Japan) was available, it was used instead of a microcatheter. Channel dilator catheters have recently become more popular in Japan due to changes of reimbersument by the national health scheme. After insertion of the antegrade guidewire, a balloon was inserted for dilation of the CTO. Then a stent was placed to maintain an adequate lumen. Antiplatelet therapy with aspirin (100 mg/day) and ticlopidine (200 mg/day) or clopidogrel (75 mg/day) was started at least 3 days before PCI and was continued subsequently.

### Statistics

Discrete variables are shown as percentages and were compared by using Fisher's exact test. Continuous variables are expressed as the mean ± SD and were compared by Student's *t*-test. In all analyses, *P* < 0.05 was considered to be statistically significant.

## RESULTS

### Number of Patients Treated for CTO (Fig. 2)

The number of patients treated for CTO has increased gradually to 200–250 annually. In particular, the number of foreign patients has increased and now exceeds that of Japanese patients. Retry cases have also increased to account for 25–30% of all CTO patients.

**Fig. 2:**
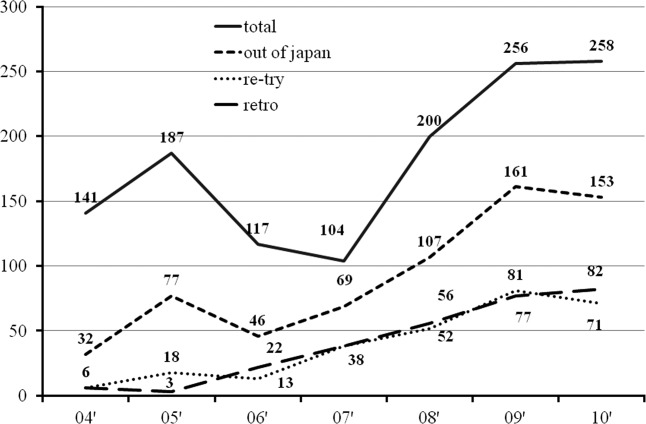
Number of PCI for CTO. Total: total number of CTO cases; retry: number of retry PCI cases; retro: number of retrograde approach cases; PCI: percutaneous transluminal coronary intervention; CTO: chronic total occlusion.

### Success Rate and the Retrograde Approach (Fig. 3)

The procedural and guidewire success rates were both 85–90%. The retrograde approach has been employed in ∼30% of CTO patients since it was fully introduced in 2006, with the guidewire success rate for this approach being ∼75–80%.

**Fig. 3:**
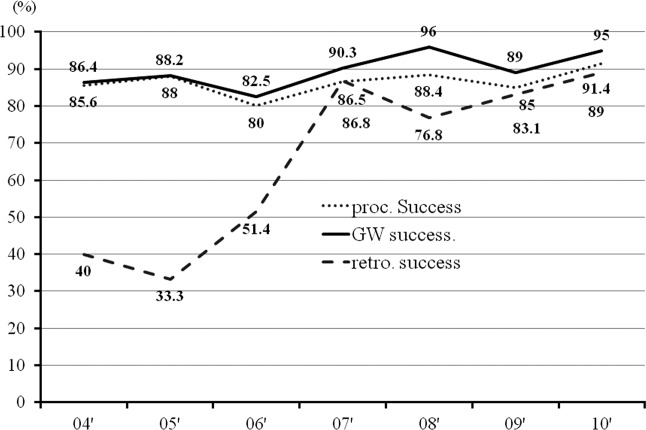
Success rate and retrograde approach for CTO. Pro. Success: procedural success rate; GW success: guidewire crossing success rate; retro success: Success rate of the retrograde approach.

### Outcome of the Retrograde Approach (Fig. 4)

The detailed outcome of the retrograde approach was investigated in 281 patients. The guidewire success rate was 81.1% and the CTO was successfully crossed in 66.5% of the patients in whom retrograde wiring was successful. The antegrade approach was tried a second time in 34 of 57 patients in whom retrograde insertion of a guidewire was unsuccessful, but the guidewire success rate was only 50% in these patients. The retrograde approach was replaced by the kissing wire technique (KWT) in 126 of the 281 patients, and the antegrade guidewire successfully crossed the CTO in 88 of them (70%). After retrograde crossing of the CTO with a guidewire, a balloon catheter was successfully passed through the collateral channel in 61 patients. The balloon catheter successfully crossed the CTO retrogradely in 48 patients, with combined use of the CART and reverse CART techniques in 22 and 21 of these patients, respectively. Corsair catheters were used in 83 patients in the later part of the series, with crossing of the CTO being successful in 63 of them. The overall procedural success rate was 79.7% (224/281 patients).

**Fig. 4:**
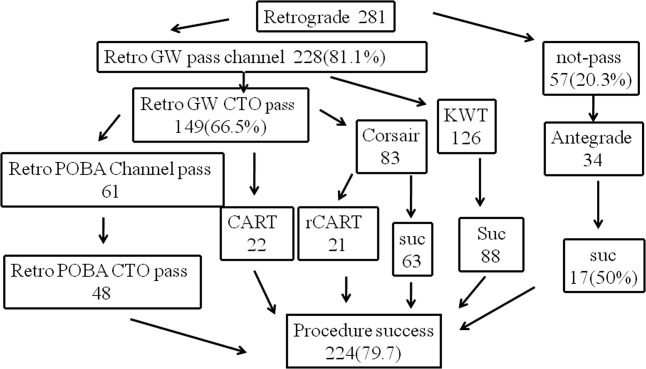
Flow chart of the retrograde approach. KWT, kissing wire technique; POBA, plain old balloon angioplasty; CART, controlled antegrade retrograde tracking technique; r-CART, reverse controlled antegrade retrograde tracking technique.

### Comparison of the Outcome Between the Early and Later Periods

Patients treated by the retrograde approach were classified into two chronological groups, which were those treated before introduction of the Corsair catheter (earlier period: 2004–2007) and after its introduction (later period: 2008–2010).

1. The retrograde approach was attempted in 67 patients during the earlier period and in 214 patients during the later period, with the percentage of all CTO cases treated retrogradely being significantly higher in the later period than the earlier period (29.2% versus 13.0%). Re-entry cases and LAD lesions were the predominant in the later period. There were no differences between the two periods with respect to risk factors, lesion sites, procedural time, volume of contrast medium used, and radiation dose. The duration of occlusion and the pathological features of the CTOs also did not differ between the two periods. An epicardial channel was more frequently selected as the collateral channel in the later period than in the earlier period (Table I).
2. The procedural success rate with the antegrade approach was 89.8% in the earlier period and 91.0% in the later period. The procedural success rate with the retrograde approach was 61.1% in the earlier period and 71.4% in the later period, while the corresponding guidewire success rates were 70.1% and 82.7%. Although both rates were higher in the later period, the differences were not significant. The CART technique was frequently used in the earlier period, whereas a Corsair catheter and the reverse CART technique tended to be more commonly employed in the later period. Channel perforation accounted for a high percentage of the complications, with no significant difference being noted between the two periods (Table II).

**TABLE I:** Backgroud of Retrograde Approach Both Period

N (retro/total)	04′–07′ 67/515	08′–10′ 214/734	*P*-value <0.001
Age	68.8+/−5.6	64.6+/−7.2	0.9
Male	56	179	0.1
Hypertension	28	118	0.4
Hypercholesterolaemia	26	120	0.2
Diabetes mellitus	22	89	0.8
MVD	54	170	0.4
EF<40%	12	50	<0.001
Re-try	28	163	
RCA	37	134	0.05
LAD	22	66	
CX	10	12	
Procedure time (min)	191.2+/−81.2	189.2+/−95.9	0.23
Contrast volume (ml)	465.6+/−154.3	481.8+/−208.4	0.16
Radiation doze (Gy)	4.7+/−3.4	4.8+/−10.4	0.33
Occlusion period 6 mon>	42	133	0.8
Unknown	25	81	1.0
Vessel size	2.7+/−1.2	2.8+/−1.6	0.5
Length	41.8+/−12.2	48.5+/−14.0	0.5
Calcium	48	204	<0.001
Angle	60	83	0.8
Unknown entry	18	56	0.9
Abrupt	3	6	0.5
Diffuse	2	5	0.8
Collateral			
Septal channel	64	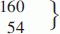	0.001
Epicardial channel	3		

**TABLE II:** Outcome fo Retrograde Approach Both Period

N (retro/total)	04′–07′ 67/515	08′–10′ 214/734	*P*-value <0.001
Antegrade success	431/480 (89.8%)	454/499 (91.0%)	n.s
Procedural success	41 (61.1%)	153 (71.4%)	n.s
Guidewire success	47 (70.1%)	177 (82.7)	n.s
Strategic variables			
Retrograde wiring	26 (38.8%)	123 (57.4%)	0.008
Kissing wire technique	33 (49.2%)	93 (43.4%)	0.41
Long shaft OTW balloon	16 (23.8%)	41 (19.2%)	0.4
Corsair catheter	0 (0%)	83 (38.7%)	<0.001
CART	12 (17.9%)	10 (4.7%)	<0.001
Reverse-CART	0 (0%)	22 (10.2%)	0.006
Complication			
CTO perforation	0 (0%)	5 (2.3%)	n.s
Channel perforation	3 (4.5%)	2 (0.9%)	n.s
Channel dissection	1 (1.5%)	5 (2.3%)	n.s
Donor vessel occlusion	0 (0%)	3 (1.4%)	n.s

### Case Report of Novel Techniques

**Fig. 5:**
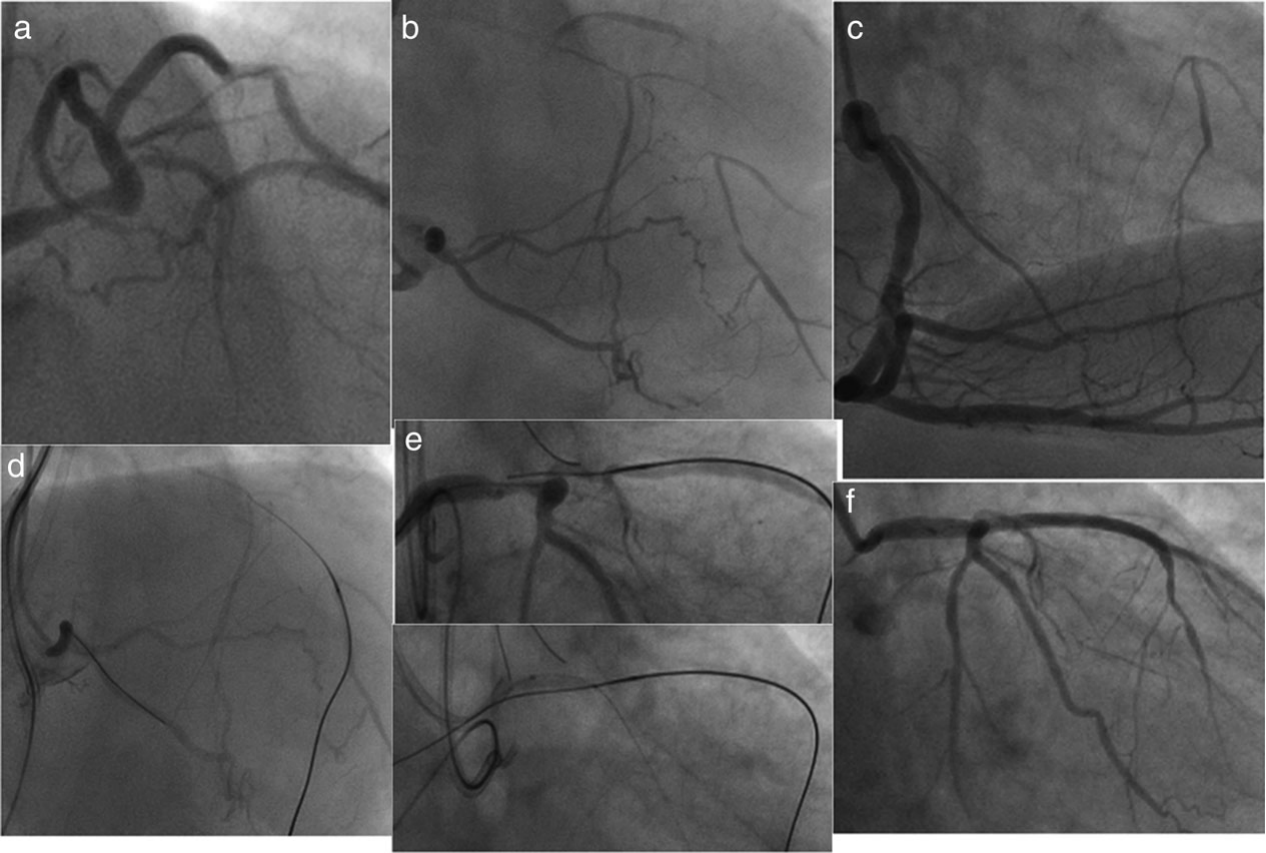
Case of retrograde approach. (a) The proximal LCA is completely occluded, but the mid-portion of the LAD receives good collateral flow. (b) Good collateral flow also reachs the middle and distal portions of the LAD via the conus branch. (c) Good collateral flow (Rentrop grade III) from the posterolateral branch of the RCA via a septal channel. (d) The Corsair catheter was advanced into the LAD and the guidewire was exchanged for Fielder XT guidewire at the distal end of the second CTO. (e) The retrograde guidewire has entered the subintimal space from the proximal end of the first CTO. (f) A 2.5 mm Ryujin balloon was inflated to 10 atm inside the LMT. By anchoring the retrograde guidewire, it was possible to advance the Corsair catheter through the CTO toward the LMT. (g) Two drug-eluting stents were deployed in order from the proximal side.

A 75-year-old man had effort angina. The proximal left coronary artery (LCA) was completely occluded, but the mid-portion of the left anterior descending artery (LAD) received good collateral flow (Rentrop grade III) from the posterolateral branch of the right coronary artery (RCA) via a septal channel. Good collateral flow also reached the middle and distal parts of the LAD via the conus branch. Occlusion of the LAD was detected at both of its proximal portion (first CTO) and its mid-portion (second CTO), with each CTO being about 30 mm in length (Fig. 5a–c). Since use of the retrograde approach was considered likely from the start of PCI, a 7F sheath and guide catheter were inserted into both femoral arteries. In addition, a 5F diagnostic catheter JR 3.5 was selectively inserted into the conus branch via the right radial artery. First, a Fielder FC guidewire (Asahi Intec Co., Japan) and a Corsair microcatheter (Asahi Intec Co.) were deployed in an attempt to cross the collateral channel. Then the Corsair catheter was advanced into the LAD and the guidewire was exchanged for a Fielder XT guidewire (Asahi Intec Co.) distal to the second CTO. Next an attempt was made to cross the second CTO, and images were obtained after injection of contrast medium through the diagnostic catheter in the conus branch to visualize the proximal end of the second CTO (Fig. 5d). When an attempt was made to cross the first CTO, however, the retrograde guidewire entered the subintimal space at the proximal end of the CTO. Because the LAD made an acute angle with the distal part of the left main trunk (LMT) and there was 75% stenosis of the LMT, it proved impossible to pass the guidewire through the guide catheter in the LCA. Therefore, to dilate the stenosed segment of the LMT and advance the Corsair catheter through the CTO, a 2.5 mm Ryujin balloon (Terumo Co., Japan) was inflated to 10 atm inside the LMT. By anchoring the retrograde guidewire, it was possible to advance the Corsair catheter through the CTO toward the LMT (Fig. 5e,f). After this, a retrograde guidewire and the Corsair catheter were led into the guide catheter in the LCA. The retrograde guidewire was exchanged for a 3 m RG3guidewire and externalization was carried out successfully. Using the 3 m RG3 guidewire, a 2.5 mm Ryujin balloon was inflated to dilate the CTOs, and two drug-eluting stent [3.0 × 33 mm^2^ and 2.5 × 28 mm^2^ Cypher stents (Johnson & Johnson Co., USA)] were deployed, achieving a good outcome (Fig. 5g).]

## DISCUSSION

### Indications

It has been reported that PCI is only indicated for CTO associated with myocardial ischemia 5. However, PCI that improves ischemia was recently suggested to also improve arrhythmia, prevent ventricular remodeling, and create useful collateral channels by restoring the patency of the CTO vessel [6,7]. Since an association between successful CTO revascularization and better long-term prognosis has been suggested, albeit against unsuccessful revascularization rather than medical therapy, the more optimal comparison. Similar results have also been obtained in patients with impaired myocardial viability [8,9]. The improved outcome may be achieved because vessel prevents cardiogenic shock by providing collateral blood supply if occlusion of other branches occurs in the future [10]. In the past, a primary success rate as low as 60–70% and a high incidence of complications have posed problems with respect to performing PCI for the treatment of CTO [10, 11]. According to data from the multicenter CTO registry in Japan, however, recent improvements of the devices and procedures for CTO have increased the primary success rate to about 90% and the incidence of coronary artery perforation has declined to only 0.3% [12]. This suggests that management of CTO by PCI has a promising future. Failure of the antegrade approach during PCI is the typical reason for attempting the retrograde approach to a CTO. Our results show that use of the retrograde approach has increased year after year, with the most common reason for selecting it being an unsuccessful initial antegrade attempt at revascularization. The retrograde approach has recently been employed in 25–30% of CTO patients. It may be indicated for patients with a poorly defined entry point and those in whom application of the antegrade approach is considered to be difficult because of severe tortuosity or calcification. The LAD, RCA, and the left circumflex artery (CX) are all considered to be target vessels for treatment of CTO and it is revascularization, if possible, because the long-term prognosis is significantly better for patients with successful revascularization [13].

### Techniques for the Retrograde Approach

When the retrograde approach is applied, passing the guidewire through a collateral channel is essential. Septal channels provide excellent visibility and are often relatively straight. In addition, these channels are located between the right and left ventricles so that blood flows into the ventricular cavity in the event of dissection or perforation with a guidewire, making such events relatively safe. Although the thick-walled epicardial channels also provide excellent visibility, these vessels are often very long and tend to have sharp curves. Moreover, if a guidewire causes perforation, massive bleeding can result in cardiac tamponade. For these reasons, a septal channel was selected in most of our patients.

Analysis of our results showed that the early success rates of both the antegrade and retrograde approach have been almost constant (85–90%) over the past few years, which is presumably ascribable to a recent increase of retry cases. In patients in whom the initial attempt via the antegrade approach is unsuccessful, the retrograde approach is often selected as a second option, and the CTO lesions of such patients are often calcified, hard, and complex.

The following reports on the retrograde approach have been published by other authors. Di Mario et al. 14 achieved a success rate of 76.5% with the retrograde approach in 17 patients, while Saito 15 reported guidewire and procedural success rates of 82 and 84%, respectively, in 45 patients. In addition, the Toyohashi Heart Center [16] reported guidewire and procedural success rates of 75.2% and 65.6%, respectively, in 157 patients treated by the retrograde approach. Moreover, the Euro CTO Club [17] reported an early success rate of 83.4% with the retrograde approach, but their series had a low proportion of retry cases (31.4%).

The transfemoral approach with at least a 7F guide catheter is recommended when PCI is performed via the retrograde approach. It is also recommended that the guide catheter should be short enough (∼90 cm) to minimize the distance to the contralateral side.

A guidewire was successfully passed through the collateral channel in 81.1% of our patients. In recent years, Fielder FC guidewires have commonly been used, but Fielder XT guidewires and others have also been selected. Fielder XT guidewires have a tapered tip (0.009 inches) and may be more suitable for passing through thinner collateral channels. The Fielder XTR guidewire (Asahi Intec Co., Ltd.; Japan) was developed recently. Its tip size is 0.010 inches, which is 0.001 inches larger than the tip of the Fielder XT guidewire, but coating as far as the tip has improved its lubrication and torque.

However, the tapered tip means that this type of guidewire lacks flexibility, so it is more likely to cause dissection at bends in the channel. Recently, the Sion Blue guidewire (Asashi Co., Japan) with excellent trackability has been developed for collateral channels, and it has become a popular option for channel manipulation.

The procedural success rate was low when the antegrade approach was tried again after failure of the retrograde approach. This low success rate was presumably related to initial failure of the antegrade approach in these patients and it seems that a satisfactory outcome is hard to obtain when the antegrade approach has to be retried because of failure with the retrograde approach.

After achieving passage through a collateral channel, a rather soft guidewire was initially used for retrograde crossing of the CTO, with the Fielder XT guidewire often being the first choice. If this guidewire failed to cross the CTO, it was replaced by a more rigid wire, such as one from the Miracle series. Recently, the Ultimate bro (Asahi Co, Japan) has often been selected as the second choice guidewire instead of a Miracle guidewire. In this situation, the guidewire success rate was only 66.5%. If retrograde crossing of the CTO was unsuccessful, the KWT was tried next. That is, an antegrade guidewire was advanced from the target vessel proximal to the CTO into the distal true lumen while using the retrograde guidewire as a landmark. After a retrograde guidewire has crossed the CTO, it is necessary to advance a balloon catheter or microcatheter to the distal part of the target vessel. This often proves difficult, and our success rate was low. Inserting the catheter into the distal part of the occluded vessel was often difficult because of blockage at bends in the collateral channel. This problem may be overcome by the septal dilation technique, in which the collateral channel is dilated with an over-the-wire (OTW) balloon inflated to a low pressure. Even when this method was applied, however, the CTO was successfully crossed with a retrograde balloon in only about 40–50% of our patients. These results suggest that a balloon catheter cannot necessarily cross CTO lesions with severe calcification or tortuous curves, even if a guidewire has been successfully advanced via the retrograde approach. However, the success rate is improving because of the recently developed Corsair channel dilator (Asahi Intecc Co., Japan). In our series, the success rate for crossing the CTO with a Corsair catheter was a high 76%, suggesting that the success rate of the retrograde approach will improve further if use of channel dilators becomes standard. If the retrograde guidewire enters a false lumen at the CTO and therefore fails to cross it, the guidewire should be advanced into the true lumen by the CART technique, which involved advancing a balloon retrogradely into the false lumen along the guidewire and inflating it to create a communication with the true lumen [18]. Alternatively, the reverse CART technique can be used, in which a balloon is inserted antegradely into the false lumen and inflated [15] (Fig. 1).

The catching retrograde guidewire technique [19, 20] is also available. In this technique, a retrograde guidewire is advanced into a guide catheter on the side of the CTO, anchored there by inflating an antegrade balloon in the guide catheter, and pulled across the CTO using a retrograde long shaft balloon. If a balloon catheter can cross the CTO, externalization is then performed. That is, a microcatheter is advanced into an antegrade guide catheter, after which the retrograde guidewire is exchanged for a 3 m guidewire using the microcatheter and then is led out of the body through the contralateral sheath. The new RG3 guidewire was specially developed for externalization and it has a very smooth coating to facilitate this procedure.

Making full use of these techniques, we achieved an overall procedural success rate of 79.7% for the retrograde approach.

According to Thompson et al. [21], the procedural success rate of PCI for CTO was significantly higher when the interventionists had experience with various techniques, including the retrograde approach, then when the interventionists were less experienced (75.2% vs. 58.9%). Their results also suggest the utility of our method of handling CTO, in which various techniques and devices are employed.

### Complications of the Retrograde Approach

Among the complications encountered with the retrograde approach, the rate of channel perforation was higher during the early period, but there was no significant difference from the later period. Channel perforation by the guidewire is generally the most feared complication. It cannot be avoided completely, even when an experienced operator manipulates the guidewire with the greatest care. If a serious perforation occurs that influences hemodynamics, tissue embolization, and pericardial drainage are the most effective interventions [13]. Donor artery occlusion is actually a more serious problem than channel perforation. If a device is inserted into a contralateral channel during PCI, acute occlusion of the donor artery may be caused by dissection or thrombosis. Cardiogenic shock occurs in most of these patients because the ipsilateral vessel is already completely occluded. Therefore, during PCI via the retrograde approach, it is necessary to maintain an activated clotting time of at least 300 sec, to be alert to the risk of guide catheter injury to the contralateral vessel, and to frequently check the status of the contralateral vessel.

### Procedural Outcome in the Earlier and Later Periods

When the earlier and later periods were compared, use of the retrograde approach showed a significant increase along with the increase of retry cases. Release of the Corsair catheter led to a higher success rate in the later period. Tsuchikane et al. have also reported that the retrograde approach with a Corsair catheter achieves excellent result, with a procedural success rate of 98.9% 22. In the later period of the present study, use of the Corsair catheter made it easy to switch to the reverse CART technique if the CTO was not crossed by the retrograde approach. This allowed us to establish a standardized strategy for the retrograde approach to CTO.

## CONCLUSIONS

In 281 patients treated for CTO by the retrograde approach, chronological changes of the procedures employed and outcomes achieved were examined. Compared with the earlier period, release of the Corsair catheter in the later period made it possible to develop a standardized strategy, so that the procedural success rate and guidewire success rate improved to 71.4% and 82.7%, respectively. In addition, the incidence of complications decreased to ∼1–2%. These findings indicate that our strategy contributes to increased safety of the retrograde approach.
